# Genome-Wide Identification and Expression Analysis of NHX Gene Family in *Ziziphus jujuba* var. *spinosa* Under Salt and Drought Stress

**DOI:** 10.3390/genes17030264

**Published:** 2026-02-26

**Authors:** Lulu Li, Xiaojun Ma, Xinhong Wang, Congcong Liu, Xiaohan Tang, Dali Geng, Xuexiang Li, Aiqin Ding, Jing Shu

**Affiliations:** 1 College of Forestry Engineering, Shandong Agriculture and Engineering University, Jinan 250100, China; z2020066@sdaeu.edu.cn (L.L.); mxjun7@163.com (X.M.); z2020017@sdaeu.edu.cn (X.W.); z2019082@sdaeu.edu.cn (C.L.); cang76858@sina.com (X.T.); arberting@outlook.com (D.G.); 13233336020@163.com (X.L.); dingaiqin0728@163.com (A.D.); 2 College of Agricultural Science and Technology, Shandong Agriculture and Engineering University, Jinan 250100, China

**Keywords:** *ZjNHX*, Na^+/^H^+^ antiporter, *Ziziphus jujuba* var. *spinosa*, expression pattern, salt stress

## Abstract

Background/Objectives: *Ziziphus jujuba* var. *spinosa* (sour jujube) is a traditional medicinal plant with remarkable tolerance to abiotic stresses, particularly salinity. However, the regulatory mechanisms underlying its salt stress tolerance remain unclear. NHX genes play a crucial role in plant adaptation to salt stress by mediating Na^+^/K^+^ transport to maintain intracellular ion homeostasis and pH balance. Although the NHX gene family has been characterized in many plant species, its functional characteristics in sour jujube have not yet been systematically investigated. Methods: In this study, using *Arabidopsis thaliana* as a reference, we identified NHX genes in sour jujube through genome-wide analysis and molecular approaches, and systematically analyzed their phylogenetic relationships, chromosomal locations, conserved motifs, gene structures, cis-regulatory elements in promoter regions, and expression patterns under abiotic stresses, particularly salt stress. Results: The results revealed the presence of eight NHX genes distributed across six chromosomes in sour jujube, which were classified into three subfamilies: Vac-class, Endo-class, and PM-class. Members within the same evolutionary clade exhibited high structural conservation in motif composition and gene architecture. Except for the PM-class, all other clades contained amiloride-binding sites (FF(I/L)(Y/F)LFLLPPI). Analysis of cis-regulatory elements indicated that the promoter regions of these genes were enriched with elements related to defense responses, stress adaptation, and phytohormone signaling, further supporting their role in plant environmental adaptation. Additionally, the qRT-PCR analysis showed that most of the *ZjNHX* genes in both roots and leaves are up-regulated by salt. Notably, *ZjNHX1* expression in roots increased approximately 40-fold within 3 h, whereas *ZjNHX2* and *ZjNHX3* were strongly induced in leaves under prolonged salt exposure. Conclusions: Taken together, this work gives a detailed overview of the *ZjNHX* genes and their important roles in response to salt stress in sour jujube. Our findings also provide a foundation for further functional characterization of this gene family.

## 1. Introduction

Soil salinization is one of the major abiotic stress factors restricting global agricultural production and sustainable development of the ecological environment. According to statistics, salt-affected soils occur in more than 100 countries, and their worldwide extent is estimated at about 1 billion ha [1]. In terms of the area of salt-affected lands, the top-ranking countries were China with 211.74 Mha [2]. Saline soils contain excessive soluble salts, mainly sodium chloride (NaCl) and sodium sulphate (Na_2_SO_4_) or other neutral salts. These salts increase osmotic pressure, diminish water availability and inhibit plant growth. Particularly, high concentrations of Na^+^ disrupt ionic homeostasis in plants, leading to osmotic stress, ionic toxicity, and oxidative damage, ultimately resulting in impaired growth and development as well as reduced yield and quality [3].

Over the course of long-term evolution, certain plants have gradually adapted to saline–alkaline environments. *Ziziphus jujuba Mill.* var. *spinosa* (sour jujube) is one such species. In China, sour jujube is an economically important fruit tree that exhibits remarkable tolerance to saline–alkaline, nutrient-poor, and drought conditions, maintaining normal physiological metabolism even in soils with 5‰ salinity [4,5]. According to Shennong’s Classic of the Materia Medica, sour jujube is documented to “calm the five viscera, lighten the body, and prolong life”. Its fruit pulp is rich in vitamins, polysaccharides, and trace elements; the buds and leaves can be processed into substitute tea and health products; the seedlings, known for their high stress tolerance, are widely used as rootstock for cultivated jujube; and the seeds (Suanzaoren) are a well-known traditional Chinese medicine, extensively applied in clinical practice for their sedative and mind-calming effects [6]. This inherent salt–alkali tolerance of sour jujube has motivated us to further investigate the underlying adaptive mechanisms.

To cope with salt stress, plants have evolved a series of complex mechanisms at both the physiological and molecular levels. Among these, Na^+^/H^+^ antiporters (NHXs) are central to maintaining osmotic balance and ion homeostasis under salt stress, regulating cytosolic Sodium (Na^+^), Potassium (K^+^), and Hydrogen (H^+^) [7]. NHX (Na^+^/H^+^ antiporter) belongs to the CPA1 superfamily and is a membrane protein composed of approximately 550 amino acid residues, exhibiting a characteristic transporter architecture [8]. The encoded protein contains a highly conserved Na^+^/H^+^ exchanger domain and typically maintains 10–12 transmembrane domains [9]. Additionally, most NHX proteins include a highly conserved 14-residue motif within the fourth transmembrane segment, represented by the consensus sequence FF(I/L)(Y/F)LFLLPPI. This region is recognized as the binding site for amiloride and its derivatives [10]. Based on their sequence identity and localization, the plant NHX family can be grouped into three distinct functional classes, which are plasma membrane (PM), vacuolar (Vac), and endosomal-associated (Endo), among which vacuolar and endosomal-associated belong to the intracellular class [8,9,11]. Different subclasses of NHX proteins exhibit significant functional differentiation.

In *Arabidopsis thaliana*, eight NHX genes have been identified. Six of these function as intracellular transporters, further divided based on their subcellular localization into two groups, vacuolar and endosome-associated [11]. The vacuolar membrane type includes *AtNHX1* to *AtNHX4*. Studies have shown that this type of gene utilizes the proton gradient generated by V-type H^+^-ATPase and H^+^-pyrophosphatase to compartmentalize excess Na^+^ from the cytoplasm into the vacuole, reducing Na^+^ toxicity and maintaining an appropriate K^+^/Na^+^ ratio in the cytoplasm, while supporting osmotic regulation through solute accumulation [11,12,13,14,15]. Recent studies have revealed that in addition to sequestering Na^+^ into vacuoles, NHX proteins may also enhance plant salt tolerance by modulating intracellular K^+^ homeostasis under saline conditions [16,17]. Furthermore, distinct functional differences exist among the vacuolar-localized isoforms *AtNHX1* to *AtNHX4*, particularly in their selectivity for transporting Na^+^ versus K^+^ [18]. *AtNHX5*/*6* belong to the endosomal type. Studies have shown that endosomal-localized NHX transporters play a critical role in maintaining intracellular ion homeostasis and pH, regulating protein processing and trafficking of cellular cargo, while also being essential for normal plant growth [11,19]. *AtNHX7*/*SOS1* and *AtNHX8* belong to the plasma membrane-localized proteins, which work synergistically with the CBL-CIPK signaling pathway to expel Na^+^ out of the cell [20,21,22]. However, this process of expelling Na^+^ out of the cell further exacerbates the imbalance of osmotic pressure and ion concentration across the membrane. Therefore, this mechanism only has transient salt resistance and cannot enable plants to have long-term salt tolerance under natural environmental conditions [23,24]. Furthermore, some researchers classify plasma membrane-localized proteins into the *SOS1* gene family. Studies have found that the NHX and *SOS1* gene families have very different evolutionary trajectories and suggest that these divergent evolutionary histories are related to the evolution of their function and cellular localization [25,26].

Multiple studies have demonstrated that Na^+^/H^+^ antiporters play a crucial role in plant salt tolerance and hold potential for genetic engineering applications. This potential is exemplified by the fact that overexpression of NHX genes, whether heterologous or homologous, can significantly enhance plant salt tolerance. For example, studies have found that when the vacuolar NHX antiporter gene *VvNHX1* from grapevine was introduced into potato, transgenic plants exhibited enhanced growth rates, tolerance, and reduced sodium ion accumulation under salt stress [27]. Moreover, overexpression of the radish *RsNHX1* gene enhanced salt tolerance in *A. thaliana*. Conversely, plants with the silence of endogenous *RsNHX1* were more susceptible to the salt stress [28], which indicates that *RsNHX1* acts as a positive regulator of salt stress resistance in radish. Moreover, transgenic tobacco plants overexpressing *IlNHX* from *Iris lactea* could compartmentalize more Na^+^ into vacuoles via increased V-ATPase activity. This mechanism maintained Na^+^/K^+^ homeostasis, improved photosynthetic capacity, and preserved plasma membrane integrity under salt stress [29]. In applied research, NHX genes have emerged as pivotal targets for developing stress-tolerant crops in cotton [30,31] and rice [32]. For example, the expression of *HtNHX1* or *HtNHX2* from *Helianthus tuberosus* improved the rice tolerance to salinity. Especially, expression of *HtNHX2* increased rice grain yield, harvest index, and total nutrient uptake under K^+^-limited salt-stress or general nutrient deficiency conditions [32]. Specifically, overexpression of NHX genes through transgenic approaches significantly enhances salinity tolerance, enabling tomato plants to sustain growth, flowering, and fruit yield under high-saline conditions. Concurrently, vacuolar Na^+^ compartmentalization mediated by these genes minimizes sodium accumulation in fruits, thereby preserving fruit quality from salt-associated impairment [33,34,35]. Collectively, these studies affirm that NHX genes are important candidate genes for the genetic improvement of crop salt tolerance, holding significant application potential.

In recent years, with the rapid development of genome sequencing technologies, the NHX gene family has been identified and functionally characterized at the whole-genome level in various plants, including crops, horticultural plants, and salt-tolerant model plants [36,37,38,39,40]. These studies have elucidated distinctive structural features, conserved motifs, and evolutionary patterns within the NHX gene family. Furthermore, accumulating evidence indicates that NHX proteins contribute not only to salt stress tolerance but also play pivotal roles in plant growth and development. Vacuolar membrane-localized NHX proteins are critical for the active uptake of K^+^ into vacuoles, regulation of cellular turgor pressure, and normal stomatal function [16,41]. Research has demonstrated that *Arabidopsis AtNHX5* and *AtNHX6* participate in the establishment and maintenance of auxin concentrations during embryogenesis and root tissue development [42]. Additionally, these proteins function within the brassinosteroid signaling pathway [43]. Collectively, NHX genes are implicated throughout the entire plant growth cycle, with their expression being significantly induced by multiple stress conditions and plant hormones.

Although sour jujube exhibits considerable salt tolerance, the underlying molecular mechanisms remain largely unexplored. Given the central role of NHX transporters in plant salt tolerance, a systematic identification and characterization of the NHX gene family in this species is essential for understanding its adaptive strategies and harnessing its genetic potential in jujube breeding. As a stress-resilient wild relative, sour jujube likely harbors unique NHX variants with distinct evolutionary and functional features. This study utilized the whole-genome data of sour jujube to comprehensively identify all members of its NHX gene family through integrated bioinformatics approaches. Systematic analyses were performed on their phylogenetic relationships, gene structures, conserved motifs, chromosomal distributions, collinearity patterns, cis-regulatory elements, and expression profiles across diverse tissues and under abiotic stresses. This study aims to elucidate the evolutionary characteristics and potential functions of the NHX gene family in sour jujube, thereby establishing a robust foundation for investigating its regulatory roles in stress responses and guiding subsequent functional validation. Ultimately, these findings will provide critical genetic resources for breeding salt-tolerant jujube varieties.

## 2. Materials and Methods

### 2.1. Genome-Wide Identification of NHX Genes in Z. jujuba var. spinosa

The genome-wide annotation data for *Z. jujuba* var. *spinosa* were retrieved from the figshare repository (https://figshare.com/s/ad5d747ccc2ccbb2b65b, accessed on 1 November 2025) [44]. A total of eight *A. thaliana* NHX protein sequences were downloaded from The Arabidopsis Information Resource (TAIR) (https://www.arabidopsis.org/, accessed on 1 November 2025). To identify candidate genes in *Z. jujuba* var. *spinosa*, BLASTP searches were conducted using the *AtNHX1*-*AtNHX8* sequences as queries, applying a strict E-value cutoff of 1 × 10^−5^ in TBtools software version 2.372 [45]. In parallel, the Hidden Markov Model (HMM) profile corresponding to the conserved NHX domain (PF00999) was acquired from the Pfam database (http://pfam.xfam.org/, accessed on 1 November 2025). This HMM profile was employed to validate candidate proteins after preliminary screening with a stringent E-value threshold of 1 × 10^−5^. Only those sequences identified by both BLASTP and HMM searches were ultimately designated as members of the *ZjNHX* gene family. Candidate sequences were submitted to the NCBI CDD and SMART databases to ensure all identified members contained the characteristic Na^+^/H^+^ exchanger (NHX) domain with default parameter settings.

### 2.2. Chromosome Localization and Protein Physicochemical Property Analysis 

Based on the genomic annotation data of *Z. jujuba* var. *spinosa*, chromosomal localization of the identified *ZjNHX* genes was visualized using TBtools software v2.372 [45]. For protein characterization, the physicochemical parameters, including theoretical isoelectric point (pI), molecular weight (MW), and instability index, were computed with the ProtParam tool on the ExPASy server (https://web.expasy.org/protparam/, accessed on 6 November 2025) [46]. Transmembrane helix regions in the *ZjNHX* proteins were then identified using the TMHMM-2.0 program [47]. Further analysis involved predicting the secondary structure composition with SOPMA (https://npsa.lyon.inserm.fr/cgi-bin/npsa_automat.pl?page=/NPSA/npsa_sopma.html, accessed on 6 November 2025) and constructing the tertiary structures via homology modeling on the SWISS-MODEL platform [48]. Finally, the subcellular localization of each *ZjNHX* protein was predicted using the Plant-mPLoc server (http://www.csbio.sjtu.edu.cn/bioinf/plant-multi/, accessed on 7 November 2025) [49].

### 2.3. Phylogenetic Tree Construction and Analysis

Multiple sequence alignment was performed using MUSCLE in MEGA v11 software under default parameters [50]. Subsequently, phylogenetic analysis was conducted based on NHX protein sequences from *Z. jujuba* var. *spinosa*, *A. thaliana*, *Cucurbita pepo*, *Camellia sinensis*, *Oryza sativa*, *Populus euphratica*, *Punica granatum*, *Solanum lycopersicum*, *Triticum aestivum*, and *Vitis vinifera*. The maximum likelihood (ML) analysis was performed using the JTT model with frequencies (+F) and incorporated a Gamma distribution with Invariant sites (G+I) to model rate heterogeneity across sites. The heuristic search for the ML tree employed the Nearest-Neighbor Interchange (NNI) method, with the initial tree generated automatically (default NJ/BioNJ). Nodal support was evaluated using the bootstrap method with 1000 replications. Finally, the phylogenetic tree was visualized and annotated using the iTOL online tool (https://itol.embl.de, accessed on 13 November 2025).

### 2.4. Conserved Motif and Gene Structure Analysis

The conserved motifs of all *ZjNHX* proteins were predicted using the MEME suite (https://meme-suite.org/meme/tools/meme, accessed on 5 November 2025), with the number of motifs expanded from the default (3) to 10 motifs [51]. Motif functional annotation was performed using the InterPro database (https://www.ebi.ac.uk/interpro/search/sequence/, accessed on 5 November 2025) [52]. To analyze the gene architecture, the exon–intron organization of each *ZjNHX* gene was determined by aligning its coding sequence (CDS) with the corresponding genomic DNA sequence using TBtools v2.372 [45]. Finally, multiple sequence alignment focusing on the conserved domains was carried out with DNAMAN v9 under default parameters.

### 2.5. Collinearity and Selection Pressure Analysis

For intra- and inter-species synteny analysis of *ZjNHX* gene family members in sour jujube, the MCScanX plugin in TBtools was employed [45] with an E-value ≤ 1 × 10^−5^, and Num of BlastHits ≥ 5. All coding sequences of syntenic gene pairs were aligned using MAFFT (https://www.ebi.ac.uk/jdispatcher/msa/mafft, accessed on 15 November 2025) with default parameters. To assess whether synonymous substitutions were saturated, substitution saturation tests were performed using DAMBE v7 with default parameters [53]. Ka, Ks, and Ka/Ks values for collinear gene pairs were calculated using TBtools. Divergence time (T) was estimated with the formula T = Ks/(2r) × 10^−6^ million years (Mya), where Ks denotes synonymous substitutions per synonymous site, and r represents the dicot-specific substitution rate of 1.5 × 10^−8^ synonymous substitutions per site per year [54].

### 2.6. Cis-Acting Element Analysis

To investigate potential transcriptional regulation, the 2 kb upstream sequences from the transcriptional start sites (TSS) of each *ZjNHX* gene were extracted from the *Z. jujuba* var. *spinosa* genome using TBtools v2.372; these were designated as the putative promoter regions. Putative cis-acting regulatory elements within these sequences were then identified through queries against the PlantCARE database (http://bioinformatics.psb.ugent.be/webtools/plantcare/html/, accessed on 24 November 2025) [55]. The identified elements, which were categorized into stress-responsive, hormone-related, plant development, and light-responsive groups, were visualized using Graphpad Prism v9 software.

### 2.7. Expression Analysis Based on RNA-Seq Data

The RNA-seq data used in this study were obtained from two publicly accessible datasets in the NCBI Sequence Read Archive (SRA) under accession numbers SRP344288 (salt stress) [5] and SRP321496 (drought stress) [56]. The salt stress dataset comprised samples from roots and leaves of sour jujube treated with 0 (control), 100, and 200 mM NaCl for 3 and 8 days [5]. The drought stress dataset included leaf samples from diploid and tetraploid plants subjected to 0 (control), 6, 12, and 48 h of drought treatment [56]. Raw reads (SRA format) were first converted to FASTQ format via the SRA Toolkit v3.0.0. Following adapter trimming and quality filtering with Trimmomatic v0.39, read quality was assessed using FastQC v0.11.9. The resulting high-quality reads were then aligned to the sour jujube reference genome employing STAR v2.7.11b, which concurrently handled genome indexing and read mapping. The expression matrix of *ZjNHX* genes was extracted using the Kallisto Super Wrapper plugin in TBtools, and expression pattern heatmaps were subsequently generated with the same software.

### 2.8. Plant Materials and Treatments

Seeds of sour jujube were collected from Zanhuang County, Hebei Province, China. Plump and uniform healthy seeds were selected and surface-sterilized with a 6–14% sodium hypochlorite solution for 15 min, followed by six successive rinses with sterile distilled water. The sterilized seeds were soaked in water for two days at 30 °C in a constant-temperature incubator to promote germination. Subsequently, the seeds were sown in seedling trays filled with a mixture of vermiculite and nutrient soil at a volume ratio of 2:1. Seedlings were cultivated under conditions of 22–25 °C with a photoperiod of 16 h light/8 h dark. At 60 days after sowing, seedlings with uniform growth were transplanted into new plug trays. After a one-week acclimatization period, all seedlings were uniformly irrigated with a 150 mM NaCl solution for salt stress treatment. Leaf and root samples were collected at 0 (control), 3, 6, 12, and 24 h after irrigation. For each time point, three biological replicates were collected, with each replicate comprising pooled samples from five individual seedlings. All harvested samples were immediately frozen in liquid nitrogen and subsequently stored at –80°C until further analysis.

### 2.9. RNA Extraction and Gene Expression Analysis

Total RNA was isolated from each sample using an RNA extraction kit (Vazyme, Nanjing, China). The concentration and purity of the RNA were assessed with a NanoDrop spectrophotometer (Thermo Fisher Scientific, Waltham, MA, USA). First-strand cDNA synthesis was then carried out with the HiScript III RT SuperMix (+gDNA wiper) (Vazyme, Nanjing, China). For quantitative real-time PCR (qRT-PCR) analysis, the ubiquitin (UBQ) gene was selected as the endogenous control. Reactions were carried out on a QuantStudio 5 Real-Time PCR system (Thermo Fisher Scientific, Waltham, MA, USA) with ChamQ Universal SYBR qPCR Master Mix (Vazyme, Nanjing, China). All reactions were run in triplicate to ensure technical reproducibility. Gene relative expression levels were calculated using the 2^−ΔΔCt^ method [57]. Statistical analyses were conducted with SPSS v27.0. Differences among treatment groups were evaluated by one-way analysis of variance (ANOVA), followed by Tukey’s honestly significant difference (HSD) post-hoc test for multiple comparisons. A probability (p) value of less than 0.05 was regarded as indicating statistical significance. All graphical representations were generated using GraphPad Prism v9. Detailed information on the primer sequences employed in this study is provided in Supplementary Table S1.

## 3. Results

### 3.1. Identification and Physicochemical Property Analysis of the ZjNHX Gene Family

**Figure 1:**
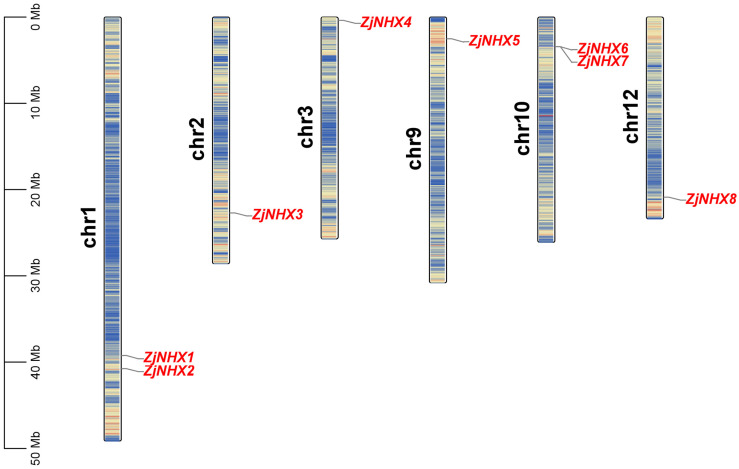
Schematic diagram of chromosomal locations of the *ZjNHX* gene family in sour jujube. All eight identified *ZjNHX* members are localized across six chromosomes, exhibiting a non-uniform distribution. The chromosome numbers are indicated on the left, and the corresponding gene names are labeled on the right.

In the genome of *Z. jujuba* var. *spinosa*, this study identified eight members of the *ZjNHX* gene family (Supplementary Table S2) distributed across six chromosomes (Figure 1). Following their linear chromosomal positions, these genes were designated *ZjNHX1* through *ZjNHX8*. Physicochemical characterization revealed significant variations in their protein properties. *ZjNHX* proteins ranged in length from 207 aa (*ZjNHX4*) to 1145 aa (*ZjNHX6*), with molecular weights spanning 22.47 kDa (*ZjNHX4*) to 126.83 kDa (*ZjNHX6*). Theoretical isoelectric points (pI) varied between 5.35 (*ZjNHX2*) and 8.75 (*ZjNHX5*), while instability indices ranged from 31.40 (*ZjNHX3*) to 42.52 (*ZjNHX8*). The grand average of hydropathy (GRAVY) values was uniformly positive (0.074–0.701), indicating the *ZjNHX* proteins were generally hydrophobic, which was consistent with the biochemical functions of NHXs as transmembrane Na^+^/H^+^ exchangers. Subcellular localization predictions indicated that *ZjNHX1*–*ZjNHX5* and *ZjNHX8* are likely vacuolar, whereas *ZjNHX6* and *ZjNHX7* are predicted to reside on the plasma membrane. Transmembrane helix predictions confirmed most proteins contain 9–12 TMs, consistent with their roles as membrane-bound transporters; however, *ZjNHX4* uniquely has only 4 TMs (Table 1).

The secondary structures of the *ZjNHX* proteins demonstrated that α-helices and random coils dominate these proteins, with proportions ranging from 43.29% to 62.30%, 23.72% to 39.32%, respectively (Supplementary Table S3). Additionally, to further analyze their structural characteristics, we predicted and visualized the three-dimensional structures using SWISS-MODEL (Supplementary Figure S1). These models revealed conserved structural architectures, providing a structural basis for functional similarity.

### 3.2. Phylogenetic Analysis

**Figure 2:**
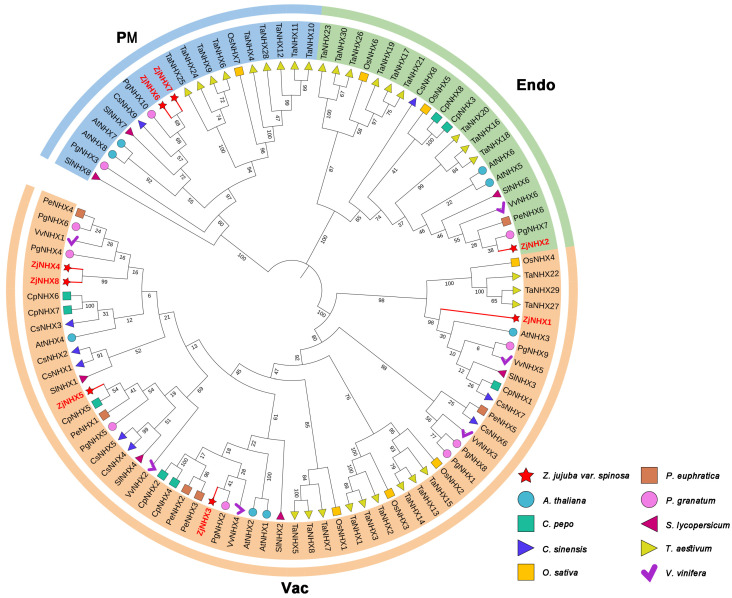
The phylogenetic tree of the NHX gene family, and sequences from *Arabidopsis thaliana* (L.) Heynh. (At), *Citrus sinensis* (L.) Osbeck (Cs), *Cucurbita pepo* (Cp), *Oryza sativa* L. (Os), *Populus euphratica* Oliv. (Pe), *Punica granatum* L. (Pg), *Solanum lycopersicum* L. (Sl), *Triticum aestivum* L. (Ta), *Vitis vinifera* L. (Vv), and *Ziziphus jujuba* var. *spinosa* (Zj). Various branch colors indicate different clades. The phylogenetic tree was constructed using the maximum likelihood method with 1000 replications.

To elucidate the evolutionary relationships of the *ZjNHX* proteins, a phylogenetic tree was constructed in this study based on 99 NHX protein sequences from *Z. jujuba* var. *spinosa* and nine other species (Figure 2, Supplementary Table S4). According to the phylogenetic tree, the 99 NHX proteins were clustered into three distinct clades, which were consistent with previous findings in *Arabidopsis AtNHX* proteins. Based on the established classification of *AtNHX* proteins, these three clades were named the vacuolar (Vac), endosomal (Endo), and plasma membrane (PM). Phylogenetic analysis revealed that the Vac clad contained the largest number of NHX proteins, followed by the Endo clad, while the PM clad comprised the fewest members. In *Z. jujuba* var. *spinosa*, the *ZjNHX* proteins were also classified into three groups. Specifically, *ZjNHX6* and *ZjNHX7* were located within the PM clade, *ZjNHX2* was positioned in the Endo clade, and the remaining five *ZjNHX* proteins (*ZjNHX1*, *ZjNHX3*, *ZjNHX4*, *ZjNHX5*, and *ZjNHX8*) were all distributed in the Vac clade. It is noteworthy that within the Vac clade, the four *ZjNHX* proteins exhibit a closer evolutionary kinship with NHX homologs from dicotyledonous plants such as *P. granatum*, *P. euphratica*, *V. vinifera*, and *C. pepo* compared with those from monocotyledonous plants like rice and wheat. This pattern is similarly observed in both the Endo and PM clades, suggesting a shared evolutionary trajectory for NHX proteins among dicotyledonous plants.

### 3.3. Exon–Intron Analysis and Motif Configuration

**Figure 3:**
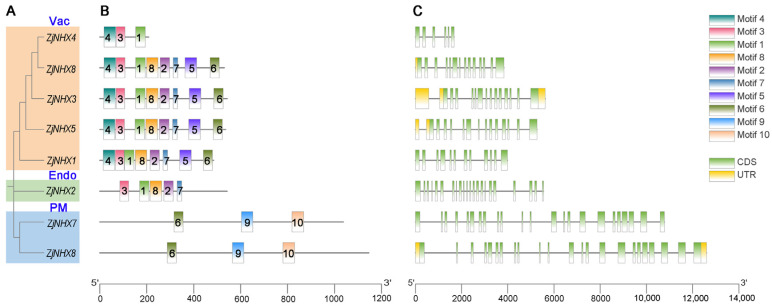
Phylogenetic tree, motif analysis, and gene structure of the *ZjNHX* gene family. (**A**) The phylogenetic tree of eight *ZjNHX* proteins is categorized into three subfamilies, distinguished by yellow (Vac-class), blue (PM-class), and green (Endo-class) background shading. (**B**) Conserved motifs in the *ZjNHX* proteins. Different motifs are represented by colored boxes, with numbers corresponding to motif types as shown in the legend on the upper right. (**C**) Exon–intron structure of *ZjNHXs*. Green rectangles represent coding sequences (CDS), yellow rectangles represent untranslated regions (UTRs), and black lines represent introns.

**Figure 4:**
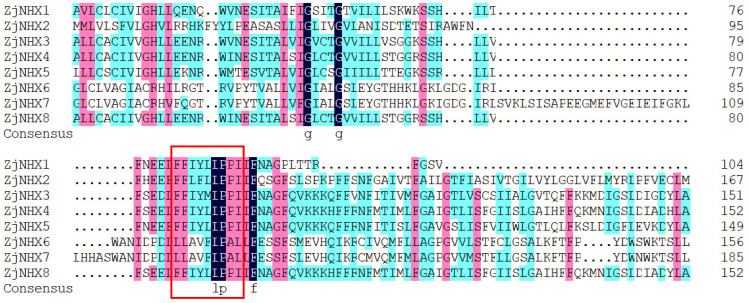
Multiple sequence alignment of amiloride-binding site (FFI/LY/FLLPPI) among *ZjNHXs*. The conserved region “FFI/LY/FLLPPI” is represented by a red box.

To provide an in-depth exploration of the structural characteristics and evolutionary relationships of the *ZjNHX* genes in *Z. jujuba* var. *spinosa*, this study performed phylogenetic analysis, conserved motif analysis, and gene structure analysis of eight *ZjNHX* proteins. This combined figure showed characteristics of the NHX gene family (Figure 3). The results of the phylogenetic tree demonstrated that all members could be divided into three major clades, which was consistent with the aforementioned phylogenetic analysis. The conservative motif distribution of *ZjNHX* proteins was consistent with the phylogenetic tree. Hence, *ZjNHX* members within the same clade exhibited similar conserved motif compositions. Specifically, motifs 4, 3, and 1 were predominantly found in the Vac clade, whereas motifs 6, 9, and 10 were mainly present in the PM clade. Functional annotation based on the InterPro database revealed that motifs 1, 2, and 3 correspond to conserved regions within the Na^+^/H^+^ exchanger domain and are widely distributed in both the Vac and Endo clades. Interestingly, motif 3 contains the amiloride-binding site (FFI/LY/FLLPPI), a characteristic feature of vacuolar NHX proteins, which was absent in the PM clade (Supplementary Figure S2). Multiple sequence alignment further corroborated this result (Figure 4). Moreover, while this site is completely conserved in four Vac-clade proteins, it harbors a single amino acid substitution in *ZjNHX3*. Gene structure analysis further revealed that genes within the same group share similarities in their genetic architecture, specifically in terms of the number and length of exons and introns. The results show that the proteins in the Vac clade mostly possess exon numbers ranging from 12 to 14. In contrast, *ZjNHX4* of this clade exhibits a markedly simplified configuration, containing only six exons, a trend that aligns with its reduced count of conserved motifs. *ZjNHX2* in the Endo clad contains 22 exons. In the PM clade, both *ZjNHX6* and *ZjNHX7* contain 23 exons, representing the most complex structure. Collectively, this gene structure analysis corroborates the classification established by the phylogenetic analysis and demonstrates that members of the same evolutionary clade exhibit a high degree of structural conservation in both conserved motif composition and exon number.

### 3.4. Analysis of Duplication Events, Collinearity, and Selection Pressure

**Figure 5:**
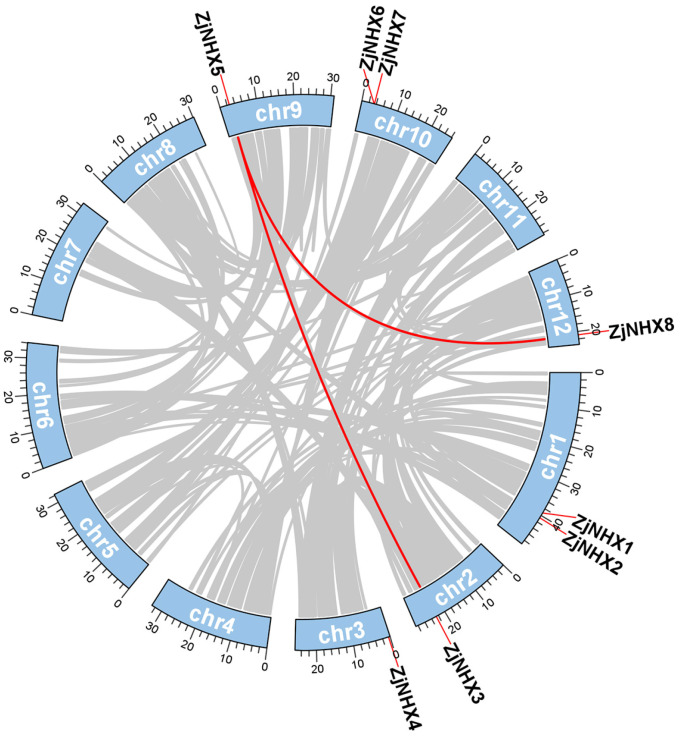
Collinearity Analysis of *ZjNHX* genes. Chromosomal distribution and intraspecific covariance analysis of the *ZjNHX* genes. Red lines connected to blue boxes represent *ZjNHX* gene pairs, and gray lines represent homologous modules in the background of the sour jujube genome.

**Figure 6:**
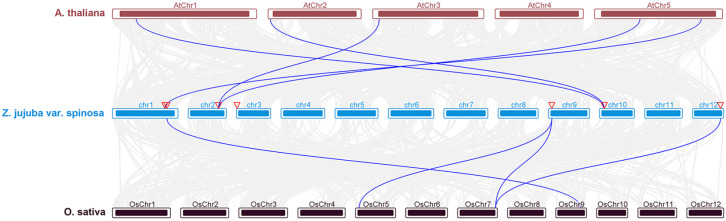
Covariance analysis of NHX across *Z. jujuba* var. *spinosa*, *A. thaliana*, and *O. sativa* chromosomes. The blue lines highlight the collinear blocks in the genomes of *Z. jujuba* var. *spinosa* and the other two plant species. The red triangles indicate the locations of the *ZjNHX* genes on the chromosome.

Gene duplication events serve as a primary driving force for genome evolution and gene family expansion. Accordingly, we analyzed the duplication events within the *ZjNHX* gene family in sour jujube. The results indicate that the evolution of the *ZjNHX* gene family was driven by various types of gene duplications (Supplementary Table S5). Specifically, *ZjNHX1*, *ZjNHX2*, and *ZjNHX4* originated from dispersed duplications, and *ZjNHX3*, *ZjNHX5*, and *ZjNHX8* are products of whole-genome duplication (WGD) or segmental duplication, while *ZjNHX6* and *ZjNHX7* arose from tandem duplications. Furthermore, we identified two syntenic gene pairs within the sour jujube genome: *ZjNHX5*-*ZjNHX3* and *ZjNHX5*-*ZjNHX8*, localized to chromosomes 9, 2, and 12, respectively (Figure 5). This spatial arrangement provides additional support for their origin via segmental duplication events. To further investigate the evolutionary constraints acting on these genes, we first assessed substitution saturation for all syntenic gene pairs. The index of substitution saturation (Iss) was significantly lower than the critical value (Iss.c, *p* < 0.05) in all pairwise comparisons, indicating no significant saturation and thus supporting the reliability of the Ks values used for subsequent divergence time estimation (Supplementary Table S6). Based on this validation, we calculated the non-synonymous (Ka) to synonymous (Ks) substitution rate ratios for these homologous pairs. The resulting Ka/Ks ratios were significantly less than 1 for all pairs (Table 2), providing clear evidence of strong purifying selection during evolution. This mode of selection likely acts to preserve the critical functional structures of the NHX gene family. Additionally, divergence time estimation revealed that these homologous pairs were formed by segmental duplications at distinct time points. The divergence between *ZjNHX5* and *ZjNHX3* occurred approximately 55.411 million years ago (Mya), whereas the divergence between *ZjNHX5* and *ZjNHX8* took place earlier, around 59.228 Mya. To further elucidate the evolutionary dynamics of the NHX gene family in *Z. jujuba* var. *spinosa*, we performed interspecies synteny analysis between sour jujube and the model plants *A. thaliana* and *O. sativa*. The results (Figure 6, Supplementary Table S7) revealed five syntenic gene pairs between sour jujube and *A. thaliana*, and four pairs between sour jujube and *O. sativa*. The higher number of syntenic pairs with *A. thaliana* likely stems from their closer phylogenetic relationship, as both are dicotyledonous plants.

### 3.5. Cis-Element Analysis of ZjNHX Promoters

**Figure 7:**
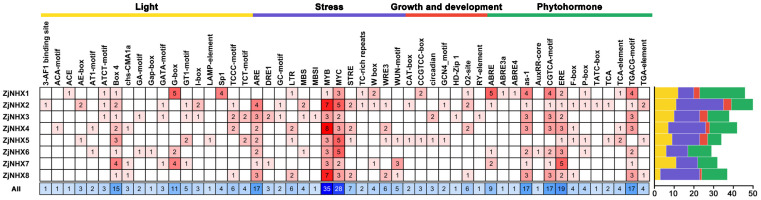
Cis-element analysis in promoter regions of *ZjNHX*. Color-coded blocks represent four functional categories of cis-elements; Yellow blocks represent light response; purple blocks represent stress response; red blocks represent growth/development regulation; and green blocks represent phytohormone response. The numerical values denote the quantity of cis-acting elements of each type.

To further explore the transcriptional regulation mechanisms and potential biological functions of the *ZjNHX* genes in sour jujube, promoter sequences spanning 2000 bp upstream of the start codon ATG for each *ZjNHX* gene were extracted and analyzed using the PlantCARE database. The results revealed a total of 52 types of cis-acting regulatory elements (CAREs), which were classified into four functional categories: light response, stress response, phytohormone response, and growth/development regulation (Figure 7; Supplementary Table S8). Light-responsive elements were the most abundant, suggesting a potentially universal role for light signaling in regulating *ZjNHX* gene expression. Beyond their well-known function in photosynthesis, these elements also integrate light signals with ABA signaling networks to modulate stress adaptation. Among the stress-responsive elements, MYB and MYC binding sites were identified 35 and 31 times, respectively, and were distributed across all *ZjNHX* promoters. These elements function as core dehydration-responsive motifs and bHLH transcription factor binding sites, both of which are critical nodes in ABA-dependent and -independent stress signaling pathways. This enrichment highlights their crucial role in mediating responses to salt, drought and low temperature [13,58]. The anaerobic induction element (ARE) was present in all members except *ZjNHX1*, implying a potential involvement of most *ZjNHX* genes in adaptation to low-oxygen environments. Within the phytohormone-responsive category, the ethylene-responsive element (ERE) was widely distributed. Elements such as “as-1” (responsive to salicylic acid and auxin), CGTCA-motif, and TGACG-motif (both responsive to methyl jasmonate) were commonly found in all promoters except in *ZjNHX5*, suggesting that these genes may be broadly involved in multiple hormone signaling pathways. Furthermore, several *ZjNHX* promoters contained elements related to growth and development, including the O2-site, CCGTCC-box, and circadian elements, hinting at their possible roles in regulating physiological processes such as seed development. Collectively, the promoter regions of sour jujube *ZjNHX* genes are enriched with diverse cis-acting elements involved in stress responses and hormone regulation, suggesting that this gene family may play a significant role in integrating various environmental signals and mediating stress adaptation processes. While these findings are descriptive and based solely on computational prediction, they provide a useful foundation for generating hypotheses about the possible involvement of this gene family in integrating environmental signals and mediating stress adaptation. Experimental validation will be necessary to confirm the actual regulatory functions of these elements.

### 3.6. Expression Analysis of ZjNHXs Based on Transcriptomic Data Under Drought and Salt Stress

**Figure 8:**
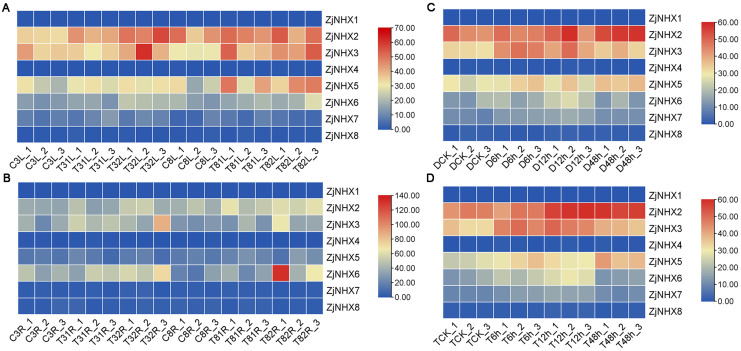
Heatmap of *ZjNHX* expression under salt and drought stress. (**A**) Expression of *ZjNHXs* in leaves after 3 and 8 days of treatment with 0, 100, and 200 mM NaCl. L, leaf; C3, 3 days of treatment with 0 mM NaCl; T31, 3 days of treatment with 100 mM NaCl; T32, 3 days of treatment with 200 mM NaCl; C8, 8 days of treatment with 0 mM NaCl; T81, 8 days of treatment with 100 mM NaCl; T82, 8 days of treatment with 200 mM NaCl. (**B**) Expression of *ZjNHXs* in roots after 3 and 8 days of treatment with 0, 100, and 200 mM NaCl. R, root; The meanings of C3, T31, T32, C8, T81 and T82 are the same as those in Figure (**A**). (**C**) Expression of *ZjNHXs* in leaves of diploid sour jujube under drought stress at 0, 6, 12, and 48 h. (**D**) Expression of *ZjNHXs* in leaves of autotetraploid sour jujube under drought stress at 0, 6, 12, and 48 h. 1–3 represent the three biological replicates.

To elucidate the functional divergence of *ZjNHX* genes under abiotic stress, we analyzed RNA-seq data to characterize their expression profiles under salt and drought (Supplementary Tables S9–S11). Transcriptome data revealed distinct response patterns of different *ZjNHX* genes to salt stress (Figure 8A,B). Specifically, *ZjNHX2*, *ZjNHX3*, *ZjNHX5*, and *ZjNHX6* were up-regulated in both roots and leaves after 3 and 8 days of exposure to 100 mM and 200 mM NaCl. Higher expression levels were observed under 200 mM NaCl compared to 100 mM at the same time points, indicating a concentration-dependent response to salt stress. In contrast, *ZjNHX1*, *ZjNHX4*, *ZjNHX7*, and *ZjNHX8* did not show significant changes in expression under salt stress. Under drought stress, five *ZjNHX* genes (*ZjNHX2*, *ZjNHX3*, *ZjNHX5*, *ZjNHX6*, and *ZjNHX7*) exhibited up-regulation in diploid and autotetraploid sour jujube (Figure 8C,D). Among them, the relative expression of *ZjNHX2* continuously increased with prolonged stress duration, peaking at 48 h. *ZjNHX3*, *ZjNHX6*, and *ZjNHX7* showed an initial up-regulation followed by a decline. *ZjNHX5* displayed a fluctuating expression pattern throughout the drought treatment. In contrast, *ZjNHX1*, *ZjNHX4*, and *ZjNHX8* did not exhibit significant expression changes during the 48 h drought period. The distinct expression patterns of *ZjNHX* genes in sour jujube under salt and drought stress indicate their diverse functional roles.

### 3.7. Expression Analysis of ZjNHX Genes Under Salt Stress

**Figure 9:**
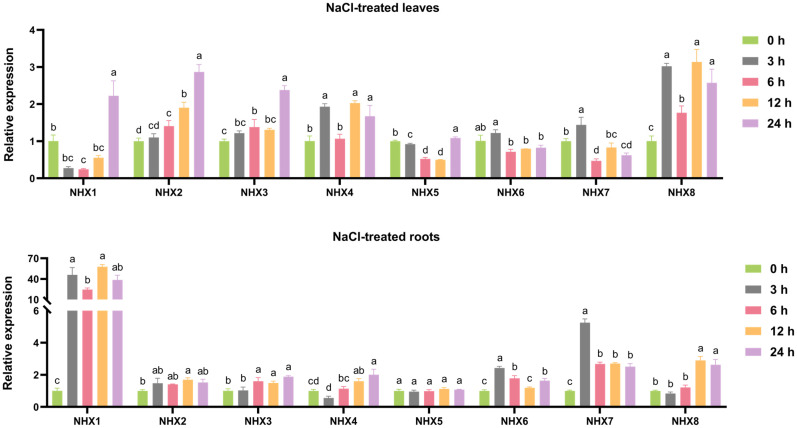
Relative expression levels of *ZjNHX* genes in the leaf and root of sour jujube seedlings subjected to 150 mM NaCl for 0, 3, 6, 12, and 24 h. Expression of the *ZjNHX* genes was normalized to that of *UBQ* and shown relative to the expression at 0 h. The 2^−∆∆Ct^ method was used to calculate the expression levels of *ZjNHX* genes at different times. Data are from three biological replicates (±SD). Statistical differences were shown by different letters at the *p* < 0.05 level according to one-way ANOVA followed by Tukey’s HSD test.

Previous studies have confirmed that salt treatment can induce the expression of NHX genes, which play a crucial role in enhancing salt tolerance in various plants. Therefore, to further understand the potential functions of *ZjNHX* genes in response to salt stress, we analyzed their expression in roots and leaves within 24 h after treatment with 150 mM NaCl, using 0 h as the control (Figure 9). RT-qPCR analysis revealed that the relative expression levels of most *ZjNHX* genes were up-regulated under salt stress. Furthermore, these genes exhibited significant temporal and spatial specificity in roots and leaves. In the roots, the expression levels of *ZjNHX1*, *ZjNHX6*, and *ZjNHX7* were rapidly up-regulated within 3 h of salt treatment. Notably, the differential expression of the *ZjNHX1* gene was the most pronounced, with its expression level increasing approximately 40-fold compared to the control group within 3 h of salt stress, and it remained at a high level at 24 h. In contrast, *ZjNHX4* and *ZjNHX8* showed an upregulating trend within 12 h of salt treatment, while the expression of *ZjNHX2*, *ZjNHX3*, and *ZjNHX5* was not significantly altered, maintaining stable levels throughout. In contrast, the relative expression level of *ZjNHX1* in leaves initially decreased and then increased, peaking at 24 h with an approximately 2-fold increase. Interestingly, transcriptomic data indicated that *ZjNHX1* exhibited almost no expression after 3 and 8 days of salt stress. It is therefore hypothesized that *ZjNHX1* expression is significantly up-regulated in response to salt stress over a short period, particularly within one day, with a more pronounced response in roots compared to leaves. Meanwhile, the expression levels of the *ZjNHX2* and *ZjNHX3* genes in leaves showed a gradual increasing trend with prolonged salt stress duration, which is consistent with the transcriptomic data. In contrast, *ZjNHX4*, *ZjNHX7*, and *ZjNHX8* exhibited a fluctuating expression pattern in leaves, characterized by an initial increase, followed by a decrease, and then another increase. These findings suggest that different *ZjNHX* gene members possess distinct response mechanisms to salt stress. The observed differential expression patterns may reflect their divergent functional roles in the salt stress response.

## 4. Discussion

The core function of the NHX gene family is to maintain cellular ion homeostasis via Na^+^/H^+^ antiport activity. Consequently, NHX proteins play pivotal roles in regulating intracellular pH, maintaining Na^+^/K^+^ balance, facilitating plant adaptation to osmotic stress, and enhancing salinity tolerance by promoting excess Na^+^ sequestration or efflux [9,18]. The number of the *NHX* gene family in most plants ranges from 7 to 35. For instance, eight *AtNHX* genes have been identified in *A. thaliana*, seven *LjNHX* genes in honeysuckle [59], ten in pomegranate [37], and thirty-five *MsNHX* genes in alfalfa [58]. In this study, eight NHX genes were identified from sour jujube. These variations in NHX gene numbers among plant species are likely attributable to gene duplication or loss events occurring in different NHX subfamilies during evolution.

Phylogenetic analysis classified the eight *ZjNHX* proteins into three clades: Vac (five members), Endo (one member), and PM (two members). The predominance of Vac-clade members is a feature shared with several other plants (e.g., *A. thaliana* [11], *T. aestivum* [60] and *Pilocarpus microphyllus* [26]) and is consistent with the hypothesis that vacuolar NHX isoforms contribute prominently to cellular ion homeostasis. In model species, phylogenetic classification generally correlates with subcellular localization. In the present study, however, an exception was observed: although *ZjNHX2* clustered within the endosomal clade, in silico tools predicted its localization to the vacuole. Similar inconsistencies between phylogenetic placement and computational predictions have been documented in other plant species, including pomegranate [37], amaranth [61], and tomato [62]. These discrepancies likely reflect the inherent limitations of current prediction algorithms when applied to complex membrane proteins such as NHX transporters [63]. Therefore, the precise subcellular localization of *ZjNHX2* in sour jujube requires further experimental validation.

The gene structures and motif compositions within the *ZjNHX* family are highly conserved among members of the same clade, supporting the phylogenetic classification. In sour jujube, exon numbers range from 6 to 14 in the Vac clade, 22 in the Endo clade, and 23 in the PM clade—a pattern broadly similar to that observed in poplar [64], tomato [62], and Arabidopsis [8], reflecting conserved evolutionary constraints across dicots. All identified *ZjNHX* proteins contain 9–12 predicted transmembrane domains, consistent with the typical architecture of Na^+^/H^+^ exchangers [65]. A notable structural feature is the amiloride-binding motif FF(I/L)(Y/F)LFLLPPI, historically considered characteristic of vacuolar NHX proteins [65,66]. In sour jujube, this motif is present in all five Vac-clade members and, interestingly, also in the single Endo-clade member (*ZjNHX2*). The occurrence of this site in endosomal isoforms has been documented in several other species [37,62,67] and may indicate partial functional conservation between Vac- and Endo-class transporters, though this remains speculative [18]. Within the Vac clade, *ZjNHX3* harbors a single amino acid substitution within the motif (I→M; FFIYMLPPI). Similar conservative substitutions have been reported in other plants (e.g., *Sorghum bicolor* [68]), and given the hydrophobic nature of both residues, the overall protein structure and core transport function are likely preserved. Whether this substitution influences inhibitor sensitivity, cation selectivity, or regulatory properties is unknown and warrants experimental investigation. Importantly, this variant may represent a lineage-specific feature of sour jujube, but its adaptive significance cannot be inferred without functional assays. Taken together, the conserved yet subtly divergent structural features of *ZjNHX* genes—particularly the atypical amiloride-binding motif in *ZjNHX3* and its presence in *ZjNHX2*—distinguish the sour jujube NHX family from those of model species. These observations generate specific hypotheses regarding isoform-specific transport properties and regulation, providing a focused entry point for mechanistic dissection in this stress-tolerant woody perennial.

Consistent with findings in other plant species [36], the *ZjNHX* genes in sour jujube exhibited distinct spatiotemporal expression patterns under salt stress. RT-qPCR analysis revealed that most *ZjNHX* members were up-regulated upon NaCl treatment, with particularly rapid and pronounced induction in roots. Notably, *ZjNHX1* expression increased approximately 40-fold within 3 h and remained elevated at 24 h; *ZjNHX7* and *ZjNHX8* showed moderate up-regulation at early time points (5-fold and 2-fold, respectively). Similar early, strong root induction of vacuolar-type NHX genes has been documented in salt-tolerant accessions of *Medicago truncatula* [69], soybean [70], and rice [71]. Although such expression kinetics are often interpreted as evidence of vacuolar Na^+^ sequestration in roots—a well-characterized function of Vac-class NHX orthologs in model species [13,18]—the present data do not directly demonstrate this mechanism in sour jujube. The observed up-regulation is consistent with, but not proof of, a role in restricting Na^+^ translocation to shoots. Direct evidence, such as ion content analysis, subcellular localization, or transgenic manipulation, is required to establish whether the rapid induction of *ZjNHX1*, *ZjNHX7*, and *ZjNHX8* indeed contributes to vacuolar compartmentalization and whole-plant salt tolerance. Nevertheless, the strong, tissue-specific responsiveness of these genes positions them as high-priority candidates for future functional studies aimed at dissecting the molecular basis of salt adaptation in sour jujube. Their expression profiles, together with the genomic resources provided here, offer a focused entry point for hypothesis-driven investigation in this stress-tolerant woody perennial.

Leaf-expressed *ZjNHX* genes also displayed salt-responsive expression patterns, with distinct temporal dynamics. *ZjNHX1* showed transient upregulation in leaves within 24 h of stress onset (~2-fold), but transcriptome data indicated negligible expression at later time points (3 and 8 d). In contrast, *ZjNHX2* and *ZjNHX3* exhibited progressive, sustained upregulation under prolonged salt exposure, with higher transcript levels under 200 mM NaCl than under 100 mM NaCl. These divergent kinetics suggest that different *ZjNHX* members may contribute to salt responsiveness over different time scales. Comparable leaf expression patterns have been reported in sugar beet (*BvNHX3*–*5*) [72] and pomegranate (*PgNHXs*) [37], indicating that delayed but sustained upregulation of certain NHX isoforms in photosynthetic tissues is not unique to sour jujube. In model species, such expression changes have been associated with vacuolar Na^+^ compartmentalization, which is thought to alleviate Na^+^ toxicity in chloroplasts and contribute to osmotic adjustment [29,73]. However, whether the observed upregulation of *ZjNHX2* and *ZjNHX3* reflects analogous physiological roles in sour jujube remains correlative and requires direct experimental testing (e.g., ion localization, transgenic approaches). Taken together, the tissue- and time-dependent expression profiles of *ZjNHX* genes under salt stress are consistent with the hypothesis that sour jujube employs spatially and temporally coordinated transcriptional responses to salinity.

## 5. Conclusions

This study presents the first genome-wide identification and characterization of the NHX gene family in sour jujube. Eight *ZjNHX* genes were identified and classified into three subfamilies based on phylogeny, conserved motifs, and gene structures. Promoter analysis revealed an enrichment of stress- and hormone-responsive cis-regulatory elements, suggesting that these genes may be transcriptionally regulated under adverse conditions. Expression profiling by both transcriptome analysis and qRT-PCR demonstrated that several *ZjNHX* members are differentially regulated under salt stress in a tissue-specific and time-dependent manner, with *ZjNHX1*, *ZjNHX2*, and *ZjNHX3* emerging as strong candidates for further functional studies. Nevertheless, the comprehensive data presented here provide a valuable genomic resource and a foundation for hypothesis-driven functional dissection of the NHX family in sour jujube and related woody perennials.

## Figures and Tables

**Table 1:** The information of physicochemical properties of the *ZjNHX* family proteins.

Gene Name	Gene ID	Chr Location	cds Length (bp)	Protein Length (aa)	MW (KDa)	pI	Instability Index	GRAVY	Subcellular Localization	Number of Predicted TMHs
*ZjNHX1*	ZspiChr1G00019980.1	chr1: 39234319–39238308 (−)	1455	484	54.19	6.8	39.59	0.562	Vacuole.	9
*ZjNHX2*	ZspiChr1G00021520.1	chr1: 40742775–40748317 (−)	1626	541	59.33	5.35	41.12	0.499	Vacuole.	11
*ZjNHX3*	ZspiChr2G00182240.1	chr2: 22684418–22690033 (−)	1626	541	59.52	8.14	31.4	0.591	Vacuole.	10
*ZjNHX4*	ZspiChr3G00227680.1	chr3: 370404–372079 (−)	624	207	22.47	6.13	31.46	0.701	Vacuole.	4
*ZjNHX5*	ZspiChr9G00120050.1	chr9: 2528315–2533575 (+)	1608	535	59.09	8.75	41.72	0.55	Vacuole.	11
*ZjNHX6*	ZspiChr10G00209970.1	chr10: 3421279–3433874 (+)	3438	1145	126.83	6.34	39.62	0.074	Cell membrane.	12
*ZjNHX7*	ZspiChr10G00209980.1	chr10: 3440271–3451048 (+)	3114	1037	115.37	5.95	37.02	0.195	Cell membrane.	10
*ZjNHX8*	ZspiChr12G00263150.1	chr12: 20861672–20865505 (−)	1590	529	58.61	7.71	42.52	0.484	Vacuole.	10

Notes: bp, base pair; aa, amino acid; MW, molecular weight; pI, isoelectric point; GRAVY, grand average of hydropathicity score; TMH, Transmembrane helices.

**Table 2:** Detailed information on the Ka, Ks, Ka/Ks ratio and divergence-time in sour jujube.

Seq 1	Seq 2	Ka	Ks	Ka/Ks	Divergence-Time/Mya
*ZjNHX5*	*ZjNHX3*	0.191	1.662	0.115	55.411
*ZjNHX5*	*ZjNHX8*	0.180	1.777	0.101	59.228

## Data Availability

The original contributions presented in this study are included in the article and Supplementary Material. Further inquiries can be directed to the corresponding author.

## Supplementary Materials

The following supporting information can be downloaded at: https://www.mdpi.com/article/10.3390/genes17030264/s1, Figure S1: Predicted tertiary structures of *ZjNHX* isoforms; Figure S2: Conserved motif distribution of *ZjNHX* proteins in sour jujube; Table S1: Sequences of RT-qPCR primers; Table S2: The coding sequence, protein, and promoter sequence information of *ZjNHX* retrieved from the jujube; Table S3: Secondary structure composition of *ZjNHX* protein; Table S4. Sequences used for phylogenetic tree construction; Table S5: The duplication events within the *ZjNHX* gene family in sour jujube; Table S6. Substitution saturation test for syntenic gene pairs; Table S7: Collinearity analysis among *Z. jujuba* var. *spinosa*, *A. thaliana* and *O. sativa* NHX gene families; Table S8: Detailed information about cis-acting elements of *ZjNHX* gene promoters; Table S9: Expression of *ZjNHXs* in leaves after 3 and 8 days of treatment with 0, 100, and 200 mM NaCl; Table S10: Expression of *ZjNHXs* in roots after 3 and 8 days of treatment with 0, 100, and 200 mM NaCl; Table S11: Expression of *ZjNHXs* in leaves of the diploid and the autotetraploid sour jujube under drought stress at 0, 6, 12, and 48 h.

## Abbreviations

The following abbreviations are used in this manuscript:

Vac	vacuolar
Endo	endosomal associated
PM	plasma membrane
pI	isoelectric point
MW	molecular weight
NJ	neighbor-joining
mM	millimolar
TMH	Transmembrane helices
GRAVY	grand average of hydropathy
CDS	Coding DNA Sequence
UTRs	Untranslated Regions
NHX	Na+/H+ antiporter
