# Supplementary figures and images for: Genome-Wide Identification and Expression Analysis of NHX Gene Family in Ziziphus jujuba var. spinosa Under Salt and Drought Stress
Source: Genes (Basel). 2026 Feb 26;17(3):264. doi: 10.3390/genes17030264 (PMC13026248; doi:10.3390/genes17030264)

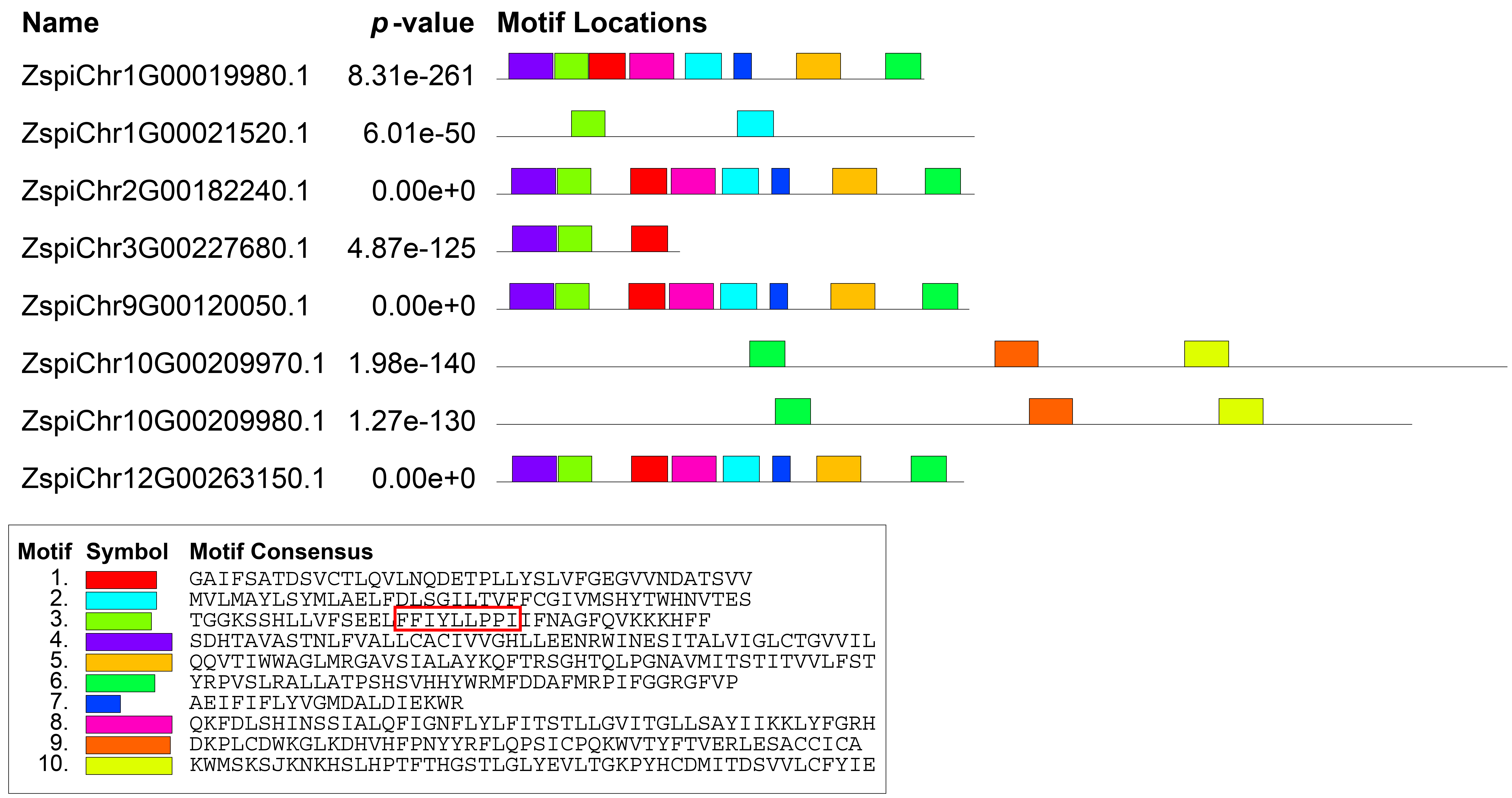

Supplement: Supplementary file 1 [file genes-17-00264-s001.zip › genes-4160914-supplementary/Figure S2. Conserved motif distribution of ZjNHX proteins in sour jujube.tif]
